# Effects of Different Commercial Formulations of Tannic Acid Added at an Equal Inclusion Rate on Intestinal Health and Microbial Flora in Yellow-Feathered Broilers

**DOI:** 10.3390/ani16132088

**Published:** 2026-07-06

**Authors:** Qingbi Gou, Yunqiu Li, Chunhui Yin, Di Yan, Hongyu Li, Juan Wang, Huali Chen

**Affiliations:** 1 College of Life Sciences and Agri-Forestry, Southwest University of Science and Technology, Mianyang 621010, China; gouqingbi@163.com (Q.G.);; 2 Mengsheng Biotechnology (Mianyang) Co., Ltd., Mianyang 621010, China; 3 New Hope Liuhe Co., Ltd., Beijing 100102, China

**Keywords:** tannic acid, dosage form, broiler chickens, gut health

## Abstract

Antibiotic restrictions require safe alternatives. Tannic acid has antimicrobial and gut-regulatory properties, but data on its formulations are limited. This study employed an equal raw material inclusion design, adding three commercial tannic acid products at 250 mg/kg to compare their effects in yellow-feathered broilers. A total of 432 broilers were assigned to four groups: control, coated (LCTA), powdered (LPTA), or granular (LGTA) tannic acid. Growth performance did not differ among groups. LPTA numerically improved ADG and reduced serum urea, while LGTA increased triglycerides. LCTA enhanced CAT and SOD activities but also elevated MDA. All treatments reduced villus height, but LPTA improved VH/CD ratio. *Claudin-1* and *Occludin* were downregulated; *ZO-1* remained stable only in LPTA. LCTA increased *IL-6*; *IL-10* and *TNF-α* decreased across treatments. LPTA appeared to maintain richness, increase diversity, and promote Bifidobacterium. The powdered form appeared to exhibit a relatively balanced profile with potential practical value, though formulation and active dose were confounded as products differed in purity (50%, 65%, 75%), resulting in different actual doses. Future studies with equalized active ingredient levels are needed to confirm formulation-specific effects.

## Simple Summary

This study evaluated three forms of tannic acid (coated, powdered, and granular) at 250 mg/kg in yellow-feathered broilers. While growth performance did not differ significantly among groups, the powdered form showed the highest average daily gain, reduced serum urea nitrogen, improved the villus height-to-crypt depth ratio, maintained *ZO-1* expression, increased cecal microbial diversity, and promoted Bifidobacterium. In contrast, the coated form elevated MDA and *IL-6*, and the granular form increased triglycerides. Overall, the efficacy of tannic acid depends on its formulation, with the powdered form providing a more balanced biological response and greater practical potential as an alternative to antibiotic growth promoters.

## 1. Introduction

Tannic acid is a naturally occurring water-soluble polyphenol widely distributed in plants, particularly in gallnuts and other tannin-rich materials. It exhibits diverse biological activities, including antioxidant, anti-inflammatory, antibacterial, and metabolic regulatory effects [1]. With increasing restrictions on antibiotic use, tannic acid has attracted considerable attention as a potential alternative in livestock and poultry production. Previous studies have demonstrated that appropriate supplementation of tannic acid can improve growth performance, enhance immune function, and modulate gut microbiota composition [2,3,4]. Recent dose–response evidence further indicates that tannic acid up to 0.075% (750 mg/kg) is tolerated and beneficial, whereas doses above 0.375% (3750 mg/kg) negatively affect growth performance in broilers [3]. Mechanistically, its biological functions are closely associated with its polyphenolic structure. On the other hand, tannic acid can interact with proteins and digestive enzymes, influencing protein degradation and amino acid utilization efficiency [5]. Furthermore, tannic acid can modulate host metabolism and immune status indirectly through alterations in gut microbiota composition and their metabolites, such as short-chain fatty acids [6,7]. However, its biological effects are dose-dependent, and excessive supplementation may induce antinutritional effects, thereby limiting its practical application [8]. Therefore, optimizing tannic acid formulations to achieve consistent efficacy while maintaining cost-effectiveness remains a critical challenge for its broader application in animal production.

From a production standpoint, dosage form is a critical factor influencing the biological efficacy of tannic acid. In practical applications, tannic acid is commonly formulated as powders, granules, or coated/microencapsulated products. Powdered formulations are easy to manufacture and cost-effective; however, their rapid dissolution may result in high localized concentrations in the gastrointestinal tract, potentially impairing palatability and inducing stress responses [2]. Previous studies have reported that dietary supplementation with 125–1000 mg/kg powdered tannic acid reduces feed intake and average daily gain in weaned piglets, thereby compromising growth performance and intestinal absorption [9]; however, other studies using different sources or dosage forms of tannic acid have reported beneficial effects on growth performance and intestinal health [4]. Granulated forms exhibit improved physical stability and handling properties, but their release characteristics remain similar to those of powders. In contrast, coated or microencapsulated formulations can modulate release behavior, reducing premature activity in the stomach and enhancing intestinal targeting [10]. For instance, conventional tannic acid shows approximately 55% release within 24 h in vitro, whereas microencapsulated formulations exhibit only about 10%, indicating a markedly prolonged release profile [4]. Microencapsulated tannic acid has been shown to improve intestinal morphology and suppress inflammatory responses via blockage of *NF-κB* in broilers [11]. Moreover, dietary inclusion of chestnut hydrolysable tannin has been shown to improve intestinal health and antioxidant capacity in broilers [12]. Collectively, these findings suggest that encapsulation systems offer effective protection and controlled release of phenolic compounds, thereby modulating their interactions with the host and gut microbiota [13,14]. Although coated or microencapsulated systems provide advantages in controlled release and targeted delivery, their complex processing results in higher production costs.

Although different dosage forms have distinct theoretical advantages, their practical efficacy varies considerably [9,15]. Rapid-release formulations may enhance immediate interactions between tannic acid and substrates but can also lead to locally elevated concentrations, whereas sustained-release or coated forms improve intestinal targeting while potentially altering interactions with the host and gut microbiota. The gut microbiota represents a primary target of dietary polyphenols and a key mediator of their biological effects [16,17]. Tannic acid has been shown to modulate microbial composition by promoting beneficial bacteria and suppressing potential pathogens, with its metabolites further influencing host energy metabolism and immune function [18,19]. However, most existing studies focus on single formulations or comparisons at different inclusion levels, and systematic evaluations under identical dosages and practical production conditions remain limited. In particular, formulation-dependent differences in metabolic regulation, intestinal function, and molecular responses remain poorly understood. Given that multiple commercial tannic acid products with different formulations are available to poultry producers, direct comparisons under identical inclusion rates are essential to inform practical decision-making regarding product selection and to optimize the cost-effective application of tannic acid in commercial poultry production.

Therefore, this study compared the effects of three tannic acid formulations—coated (LCTA), powdered (LPTA), and granulated (LGTA)—in yellow-feathered broilers at an equal commercial inclusion rate of 250 mg/kg. Through comprehensive evaluation of growth performance, serum biochemistry, intestinal morphology, antioxidant status, gene expression, and gut microbiota, we aimed to identify formulation-specific responses and provide practical guidance for the use of tannic acid products in poultry production.

## 2. Materials and Methods

### 2.1. Experimental Animals and Housing Conditions

A total of 432 healthy 1-day-old yellow-feathered broiler chickens (Pengshan New Hope Feed Co., Ltd., Meishan City, Sichuan Province, China) with similar initial body weight were used in this study. Birds were floor-reared with ad libitum access to feed and water and vaccinated according to a standard immunization program. Environmental conditions, including ventilation, temperature, and lighting, were maintained consistently in accordance with standard broiler management practices. Throughout the experiment, birds were monitored daily for behavior, fecal characteristics, and mortality, and all observations were recorded promptly. All procedures involving animals complied with the Chinese Guidelines for the Welfare and Ethical Review of Laboratory Animals and were approved by the Animal Ethics Committee of Southwest University of Science and Technology (Approval No. SWUST2024023).

### 2.2. Experimental Materials

Three hydrolyzed tannic acid products were provided by Mengsheng Biotechnology Co., Ltd. (Mianyang, China). The coated product (LCTA) was a microencapsulated hydrolyzed tannic acid prepared via spray-coating technology, containing 50% active ingredient and exhibiting sustained-release properties. The powdered product (LPTA) was a conventional powdered hydrolyzed tannic acid with a concentration of 65%. The granular product (LGTA) was a granulated hydrolyzed tannic acid with a concentration of 75%. The three products were commercial formulations; detailed information regarding carriers, coating materials, encapsulation technology, and excipients was proprietary and could not be disclosed by the manufacturer. The three products originated from the same manufacturing batch of raw material and differed only in their processing technologies (spray-coating for LCTA, conventional powdering for LPTA, and granulation for LGTA). Quality-control certificates were available for each product batch, confirming that the active ingredient content met the specified concentrations (50%, 65%, and 75%, respectively) and that the products complied with relevant feed additive quality standards.

### 2.3. Experimental Design and Diets

A completely randomized design with a single-factor arrangement was employed. A total of 432 one-day-old yellow-feathered broilers were randomly allocated to four treatment groups, with six replicates per treatment and 18 birds per replicate [20]. The control group (CON) received a basal diet, whereas the treatment groups were fed the basal diet supplemented with 250 mg/kg of coated (LCTA), powdered (LPTA), or granulated (LGTA) tannic acid, respectively. For molecular, histological, and microbiota analyses, one bird per replicate with body weight closest to the replicate mean was selected, and the replicate served as the experimental unit for all statistical analyses.

The inclusion level of hydrolyzed tannic acid products was based on product weight (250 mg/kg of complete feed, i.e., 0.025%), without correction for active tannic acid content, to allow direct comparison of the three formulations under practical production conditions. The experiment lasted 42 days. The basal diets were supplied by New Hope Group and formulated in phases according to the nutritional requirements of yellow-feathered broilers at different growth stages. The diet compositions are presented in Table 1.

### 2.4. Sample Collection and Processing

Birds were fasted for 12 h prior to sampling. At 42 days of age, one bird per replicate with body weight close to the replicate mean was selected. Blood samples were collected from the wing vein into vacuum tubes, centrifuged at 3000× *g* for 10–15 min, and the serum was collected and stored at −20 °C until analysis [21].

A 2 cm segment of the jejunum was collected and gently rinsed with ice-cold saline. The tissue was then fixed in 4% paraformaldehyde (in PBS) and subsequently transferred to 75% ethanol for storage prior to paraffin embedding and morphological analysis [22]. Adjacent jejunal samples were also collected, and the mucosa was gently scraped, aliquoted into cryovials, immediately frozen in liquid nitrogen, and stored at −80 °C until analysis.

Cecal contents were aseptically collected, aliquoted into sterile cryovials, immediately frozen in liquid nitrogen, and stored at −80 °C for subsequent analysis.

### 2.5. Measurement Parameters and Methods

Bird health status was monitored and recorded daily throughout the experiment. Birds were weighed by replicate following a 12 h fast at 21 days of age to determine initial body weight (IBW) for the 1–21 d phase, and at 42 days of age to determine final body weight (FBW). Feed intake, refusals, and wastage were recorded weekly throughout the experiment to calculate average daily feed intake (ADFI), average daily gain (ADG), and feed conversion ratio (FCR).

Average daily feed intake (ADFI), average daily gain (ADG), and feed conversion ratio (FCR) were calculated on a replicate basis for each phase [23]. Mortality was accounted for by excluding dead birds from performance calculations. The calculations were performed as follows:

ADG (g/day) = (Final body weight − Initial body weight)/(number of birds × number of days)

ADFI (g/day) = (total feed supplied − feed refusals − feed consumed by dead birds)/(number of birds × number of days)

FCR = total feed intake (kg)/total body weight gain (kg)

#### 2.5.1. Serum Biochemical Parameters

Serum biochemical parameters included total protein (TP), albumin (ALB), total cholesterol (TC), triglycerides (TG), urea nitrogen (UREA), and globulin (GLOB). All parameters were determined using an automated biochemical analyzer (BIOBASE, Jinan, Shandong, China).

#### 2.5.2. Intestinal Histology

At 42 days of age, one bird per replicate with body weight close to the replicate mean was selected and euthanized. Jejunal samples were fixed in 4% paraformaldehyde, dehydrated, embedded in paraffin, and sectioned following standard histological procedures. Sections were stained with hematoxylin and eosin (H&E) for morphological observation under a light microscope (CX41, Olympus, Tokyo, Japan).

For each section, three to five well-oriented fields were selected for measurement of villus height (VH) and crypt depth (CD), and the villus height-to-crypt depth ratio (VH/CD) was calculated [24].

#### 2.5.3. Intestinal Antioxidant Capacity

Approximately 0.1 g of jejunal mucosa was collected and homogenized with ice-cold saline at a ratio of 1:9 (*w*/*v*) using a homogenizer (Shanghai Shenlu Homogenizer Co., Ltd., Shanghai, China) in an ice bath to obtain a 10% (*w*/*v*) homogenate. The homogenate was centrifuged at 3000× *g* for 10–15 min, and the supernatant was collected for subsequent analysis.

Catalase (CAT, A007-1-1) and superoxide dismutase (SOD, A001-1-2) activities, as well as malondialdehyde (MDA, A003-1-2) content, were determined using commercial assay kits (Nanjing Jiancheng Bioengineering Institute, Nanjing, China) according to the manufacturer’s instructions [25].

#### 2.5.4. Quantitative Real-Time PCR Analysis

Jejunal mucosal samples were thawed on ice, and total RNA was extracted using the TRIzol method with RNAiso Plus reagent (Jiangsu Cowin Biotech Co., Ltd., Taizhou, China). RNA concentration and purity were assessed using a NanoDrop ND-2000 spectrophotometer (Thermo Fisher Scientific, Wuhan, China). First-strand cDNA was synthesized from qualified RNA templates according to the manufacturer’s instructions.

The qRT-PCR reaction (20 µL) contained 10 µL of 2× PerfectStart^®^ Green qPCR SuperMix, 1 µL each of forward and reverse primers (10 μM), 1 µL of cDNA template, and nuclease-free water to volume. β-actin was used as the reference gene. Amplification was performed using a real-time PCR system under the following conditions: initial denaturation at 95 °C for 30 s, followed by 40 cycles of denaturation at 95 °C for 5 s and annealing/extension at 60 °C for 30 s. A melting curve analysis was conducted to confirm amplification specificity.

All reactions were performed in triplicate, and relative gene expression levels were calculated using the 2^−ΔΔCt^ method. Primer sequences (Sangon Biotech, Shanghai, China) are listed in Table 2.

#### 2.5.5. Cecal Microbiota Analysis

At 42 days of age, following euthanasia, cecal contents were aseptically collected, immediately frozen in liquid nitrogen, and stored at −80 °C until analysis. 16S rRNA gene sequencing was conducted by Novogene Co., Ltd. (Beijing, China) using the Illumina NovaSeq platform. Paired-end sequencing was performed targeting the V3–V4 hypervariable regions of the 16S rRNA gene. Raw reads were merged, quality-filtered, and processed using QIIME2 (version QIIME2-2022.02) with the DADA2 plugin to obtain high-quality clean data. Sequences were denoised into amplicon sequence variants (ASVs), followed by taxonomic annotation against the SILVA database (version 138) and relative abundance analysis to characterize microbial community composition. Alpha diversity was assessed using Shannon and Simpson indices (diversity) and Chao1 and Observed species indices (richness), with one-way ANOVA used for group comparisons. Beta diversity was evaluated using weighted UniFrac distance, and differences among groups were tested using PERMANOVA (Adonis) with 999 permutations. Differential abundance analysis among groups was performed using LEfSe (Linear Discriminant Analysis Effect Size) with a one-against-one multi-class analysis strategy, and taxa with an LDA score threshold ≥ 2.0 were considered significantly enriched. Additionally, Wilcoxon rank-sum test and Kruskal–Wallis test were applied for pairwise and multi-group comparisons, respectively.

### 2.6. Statistical Analysis

All data were analyzed using SPSS version 26.0 (IBM Corp., Armonk, NY, USA). The pen (replicate) served as the experimental unit for all statistical analyses (*n* = 6 per treatment). For data satisfying normality (Shapiro–Wilk test) and homogeneity of variance (Levene‘s test) assumptions, parametric tests were applied: one-way analysis of variance (ANOVA) followed by Duncan’s multiple range test for post hoc comparisons. Duncan‘s test was selected for its higher statistical power in detecting differences among a limited number of treatment groups and its widespread acceptance in poultry nutrition research, with the understanding that all significant findings were interpreted with appropriate caution. For microbiome NGS data, including alpha diversity indices, beta diversity distances, and genus-level relative abundances, non-parametric tests were used: Kruskal–Wallis test for multi-group comparisons and Wilcoxon rank-sum test for pairwise comparisons. For microbiota relative abundance data, arcsin-square root transformation was applied before statistical analysis to improve normality and stabilize variance. For LEfSe analysis, an LDA score threshold of ≥2.0 was applied to identify differentially abundant taxa. Results are presented as mean ± standard deviation (SD). Differences were considered statistically significant at *p* < 0.05.

## 3. Results

### 3.1. Growth Performance

As presented in Table 3, no significant differences in growth performance were observed among treatments (*p* > 0.05). Compared with the CON group, ADFI showed a numerical increasing trend in the LCTA, LPTA, and LGTA groups. Additionally, ADG was numerically higher in the LPTA group than in the other treatments, although these differences were not statistically significant.

### 3.2. Serum Biochemistry

As shown in Table 4, compared with the CON group, the levels of total protein (TP), albumin (ALB), and globulin (GLOB) were significantly higher in the LCTA, LPTA, and LGTA groups (*p* < 0.05). Regarding lipid metabolism, there were no significant differences in total cholesterol (TC) levels among the treatment groups compared to the CON group (*p* > 0.05), but the TC level in the LCTA group was significantly higher than that in the LPTA group (*p* < 0.05). In addition, triglyceride (TG) levels in the LGTA group were significantly higher than those in the CON and LPTA groups (*p* < 0.05), while urea nitrogen (UREA) levels in the LPTA group were significantly lower than those in all other groups (*p* < 0.05).

### 3.3. Intestinal Tissue Morphology

**Figure 1:**
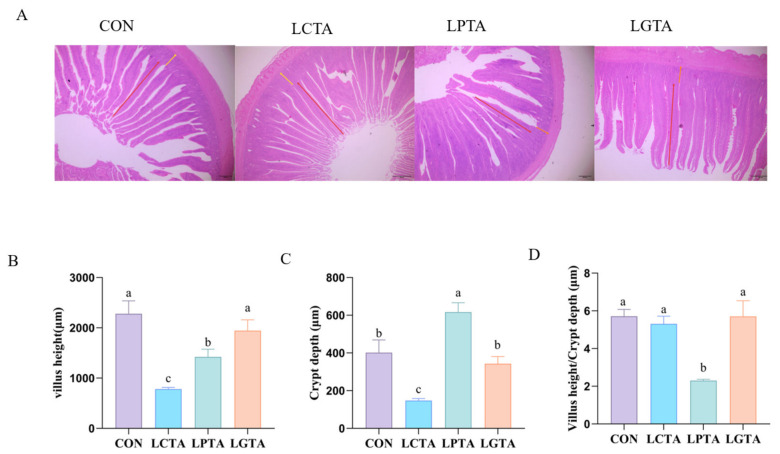
Effects of different formulations of tannic acid on jejunal morphology in yellow-feathered broiler chickens. (**A**) Representative histological images (40×; scale bar = 500 μm); (**B**) villus height; (**C**) crypt depth; (**D**) villus height-to-crypt depth ratio (VH/CD). Data are presented as mean ± SD (*n* = 6 per group). Different lowercase letters indicate significant differences among groups (*p* < 0.05, one-way ANOVA followed by Duncan‘s multiple range test), while the same letters indicate no significant difference (*p* > 0.05). Red arrows denote villus height, while orange arrows denote crypt depth.

As presented in Figure 1A, jejunal villi in the CON group exhibited normal morphology, whereas villus height was significantly reduced in the LCTA, LPTA, and LGTA groups. Compared with the CON group, villus height and crypt depth were significantly altered in the LCTA and LPTA groups (*p* < 0.05) (Figure 1B,C).

As shown in Figure 1D, the villus height-to-crypt depth ratio (VH/CD) was significantly higher in the LPTA group than in the other treatments (*p* < 0.05).

### 3.4. Intestinal Antioxidant Status

**Figure 2:**
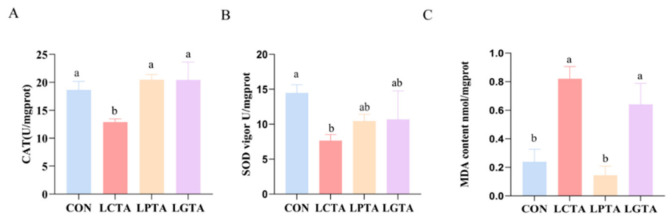
Effects of different formulations of tannic acid on jejunal antioxidant enzyme activities in yellow-feathered broiler chickens. (**A**) Catalase (CAT); (**B**) total superoxide dismutase (T-SOD); (**C**) malondialdehyde (MDA). Data are presented as mean ± SD (*n* = 6 per group). Different lowercase letters indicate significant differences among groups (*p* < 0.05, one-way ANOVA followed by Duncan’s multiple range test), while the same letters indicate no significant difference (*p* > 0.05).

As presented in Figure 2, compared with the CON group, the LCTA group showed significant alterations in CAT and SOD activities, as well as MDA levels (*p* < 0.05) (Figure 2A–C). In contrast, the LPTA and LGTA groups exhibited a trend toward increased CAT activity and decreased SOD activity (Figure 2A,B). Notably, MDA levels were numerically lower in the LPTA group than in the other treatments.

### 3.5. Intestinal Gene Expression

**Figure 3:**
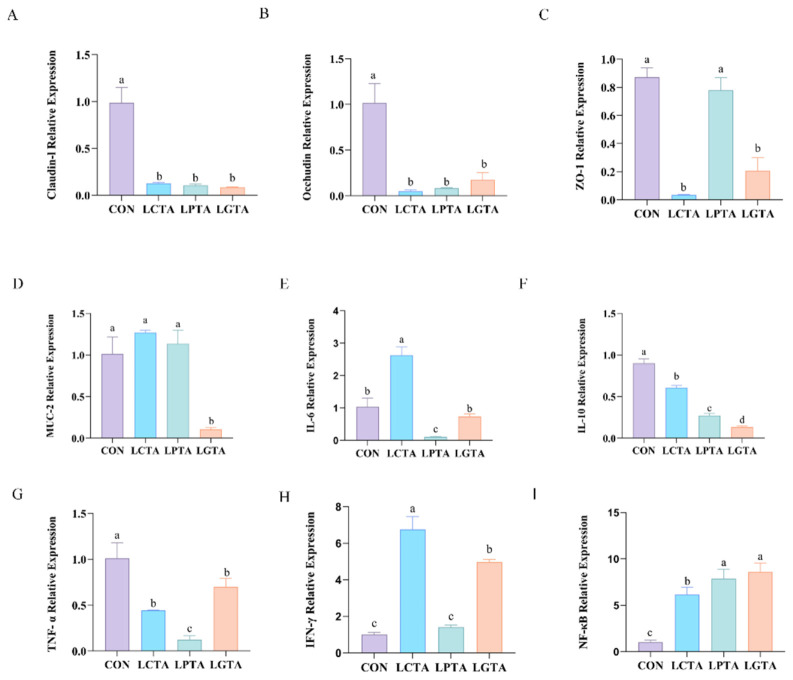
Effects of different formulations of tannic acid on jejunal gene expression in yellow-feathered broiler chickens. (**A**–**D**) Intestinal barrier-related genes: *Claudin-1*, *Occludin*, *ZO-1*, *MUC-2*; (**E**–**I**) Immune-related genes: *IL-10*, *TNF-α*, *IFN-γ*, *IL-6*, *NF-κB*. Data are presented as mean ± SD (*n* = 6 per group). Different lowercase letters indicate significant differences among groups (*p* < 0.05, one-way ANOVA followed by Duncan’s multiple range test), while the same letters indicate no significant difference (*p* > 0.05).

As presented in Figure 3, with respect to tight junction-related genes, *Claudin-1* and *Occludin* expression levels were significantly downregulated in the LCTA, LPTA, and LGTA groups compared with the CON group (*p* < 0.05) (Figure 3A,B). In addition, *ZO-1* expression was significantly reduced in the LCTA and LGTA groups (*p* < 0.05) (Figure 3C).

With respect to mucin expression, *MUC-2* levels showed a numerical increase in the LCTA and LPTA groups without statistical significance (*p* > 0.05), whereas *MUC-2* expression was significantly downregulated in the LGTA group (*p* < 0.05) (Figure 3D).

In terms of inflammatory cytokines, *IL-6* expression was significantly upregulated in the LCTA group (*p* < 0.05), whereas *IL-10* and *TNF-α* expression levels were significantly downregulated in all treatment groups (*p* < 0.05). *IFN-γ* expression was significantly increased in the LCTA and LGTA groups (*p* < 0.05). Additionally, *NF-κB* expression was significantly upregulated in all treatment groups compared with the CON group (*p* < 0.05) (Figure 3E–I).

### 3.6. Gut Microbiota

**Figure 4:**
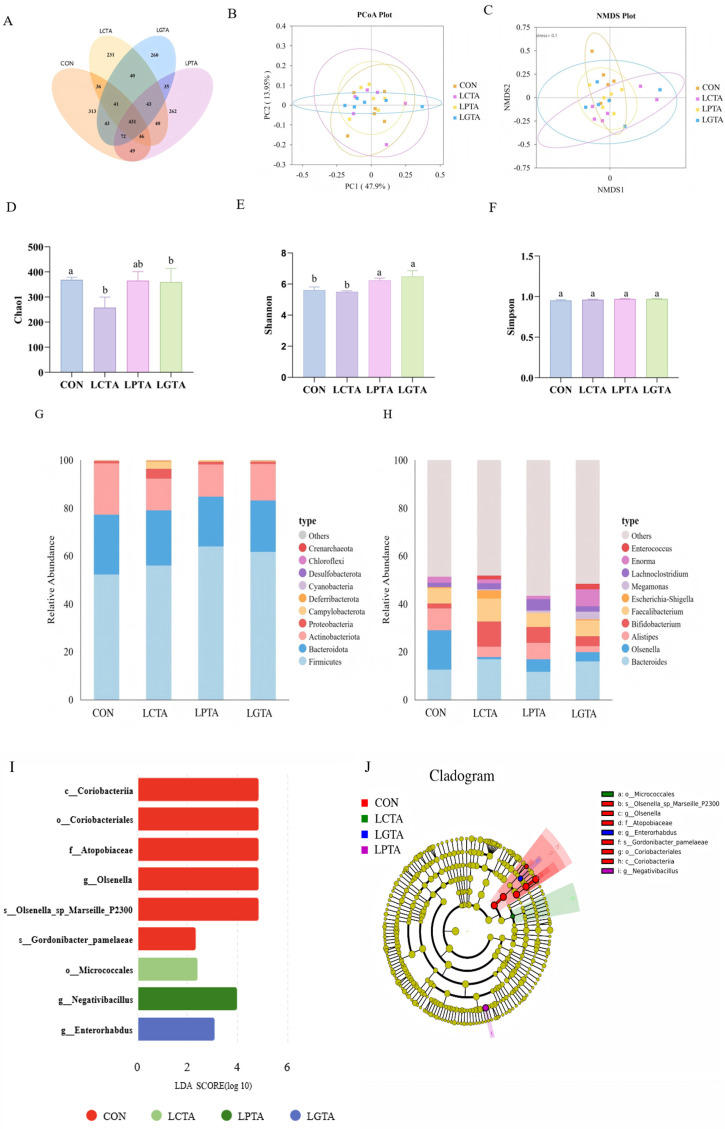
Composition of the top 10 communities by gut microbiota diversity and abundance. (**A**) Venn diagram showing shared and unique ASVs among groups; (**B**) Principal coordinate analysis (PCoA) based on weighted UniFrac distances; (**C**) Non-metric multidimensional scaling (NMDS) analysis (stress = 0.1); (**D**–**F**) Alpha diversity indices: observed species, Chao1, Shannon; (**G**) Relative abundance at the phylum level; (**H**) Relative abundance at the genus level; (**I**,**J**) LEfSe analysis: (**I**) LDA score histogram (LDA score ≥ 2.0), (**J**) phylogenetic tree of differentially abundant taxa. Data are presented as mean ± SD (*n* = 6 per group). For alpha diversity, different lowercase letters indicate significant differences among groups (*p* < 0.05, one-way ANOVA followed by Duncan’s multiple range test). Beta diversity was assessed using PERMANOVA (Adonis) with 999 permutations. LEfSe was performed with a one-against-one multi-class analysis strategy and an LDA score threshold ≥ 2.0.

High-throughput 16S rRNA gene sequencing was employed to profile the gut microbial community structure across treatments. ASV-based analysis identified 431 shared amplicon sequence variants, suggesting the existence of a core microbiota common to all groups (Figure 4A).

Beta diversity analysis revealed distinct clustering patterns among groups. Principal coordinate analysis (PCoA) based on weighted UniFrac distances explained 47.9% (PC1) and 13.9% (PC2) of the total variance, showing visual separation of microbial community structures among treatments (Figure 4B). Non-metric multidimensional scaling (NMDS) further supported this observation, with a stress value of 0.1 indicating reliable ordination and clear group clustering (Figure 4C). However, PERMANOVA (Adonis) with 999 permutations based on weighted UniFrac distances did not detect statistically significant differences among groups (R^2^ = 0.153, *p* = 0.152), suggesting that the observed clustering tendencies require cautious interpretation, potentially due to the limited sample size and within-group variability.

Alpha diversity analysis demonstrated that microbial richness (Chao1 index) was significantly reduced in the LCTA and LGTA groups compared with the CON group (*p* < 0.05), whereas microbial diversity (Shannon index) was significantly increased in the LPTA and LGTA groups (*p* < 0.05). No significant difference was observed in the Simpson index among treatments (Figure 4D–F).

At the phylum level, *Firmicutes*, *Bacteroidota*, and *Actinobacteriota* were the dominant bacterial phyla across all groups (Figure 4G). Compared with the CON group, the relative abundance of *Firmicutes* was numerically higher in the LPTA and LGTA groups, whereas *Bacteroidota* and *Actinobacteriota* showed a decreasing trend. The bacterial community composition in the LCTA group was similar to that in the CON group, but the proportion of the *Actinobacteria* phylum was slightly higher than in the CON group, and a low level of *Campylobacteria* phylum was detected. Minor phyla, including *Proteobacteria* and *Campylobacterota*, were present at low abundance across all treatments.

At the genus level, the CON group was dominated by *Olsenella* and *Bacteroides* (Figure 4H). The LCTA group showed numerically higher relative abundances of *Bifidobacterium* and *Faecalibacterium*, whereas the LPTA group was characterized by the presence of *Alistipes* and *Bifidobacterium*. In the LGTA group, *Enorma* was notably abundant.

LEfSe analysis (LDA score > 2.0) further identified group-specific microbial biomarkers (Figure 4I,J). The CON group was enriched in taxa including *Coriobacteria*, *Coriobacteriales*, *Atopobiaceae, Olsenella*, and *Olsenella_*sp*_Marseille_P2300*, as well as *Gordonibacter_pamelaeae*. The LCTA group was enriched in *Micrococcales*, the LPTA group in *Negativibacillus*, and the LGTA group in *Enterorhabdus*.

### 3.7. Functional Prediction of Gut Microbiota Based on Tax4Fun Analysis

To further explore the potential functional characteristics of the gut microbiota, Tax4Fun was applied to predict KEGG functional profiles based on 16S rRNA sequencing data. At KEGG Level 2, the predicted functional composition was mainly dominated by carbohydrate metabolism, membrane transport, replication and repair, amino acid metabolism, translation, and energy metabolism. Compared with the control group, tannic acid supplementation resulted in a lower predicted relative abundance of carbohydrate metabolism-related functions, as well as varying decreases in membrane transport, replication and repair, and amino acid metabolism-related functions. In contrast, translation- and energy metabolism-related functional categories showed increasing trends. Other predicted functional categories, including nucleotide metabolism and glycan biosynthesis and metabolism, exhibited relatively minor variations among groups.

**Figure 5:**
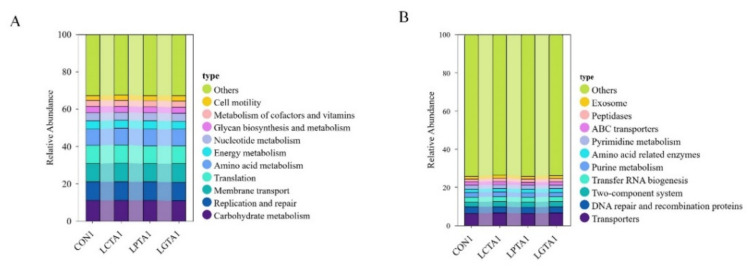
Predicted functional profiles of gut microbiota based on Tax4Fun analysis. (**A**) KEGG Level 2 functional categories; (**B**) KEGG Level 3 functional categories. Data are presented as mean relative abundance (*n* = 6 per group).

Further analysis at KEGG Level 3 showed patterns generally consistent with those observed at Level 2. The predicted abundances of transporter proteins, ABC transporters, DNA repair and recombination proteins, purine metabolism, and pyrimidine metabolism-related functions showed decreasing trends following tannic acid supplementation, whereas tRNA biosynthesis and two-component system-related functions showed increasing trends (Figure 5A,B).

Overall, Tax4Fun prediction suggested that tannic acid supplementation may influence the potential functional composition of the gut microbiota, particularly pathways related to nutrient metabolism, transport, and cellular maintenance. However, the predicted functional profiles among LCTA, LPTA, and LGTA groups showed largely comparable patterns, suggesting limited formulation-specific effects on microbial functional potential under the present experimental conditions.

## 4. Discussion

This study systematically evaluated the biological effects of three formulations of tannic acid in yellow-feathered broiler chickens. The results suggest that, although no significant differences in growth performance were observed, formulation-specific differences occurred in protein metabolism, redox status, intestinal barrier integrity, and gut microbiota modulation. However, the three products were compared at equal commercial inclusion rates (250 mg/kg), and their actual active tannic acid content differed (50%, 65%, and 75% for coated, powdered, and granular, respectively). Therefore, the observed product-specific differences may reflect both formulation characteristics and actual dose variations. Among the tested formulations, LPTA appeared to exhibit a relatively balanced biological profile under the present experimental conditions.

With respect to growth performance and metabolic responses, the present findings are consistent with previous reports indicating that low-to-moderate levels of tannic acid (≤500 mg/kg) do not markedly impair growth performance in broilers [26,27]. Although no significant differences were observed among treatments, the LPTA group showed a numerically higher average daily gain, accompanied by significantly reduced serum urea nitrogen (UREA) levels compared with the other groups. As an indicator of protein catabolism, reduced UREA levels generally indicate enhanced nitrogen utilization efficiency and/or decreased amino acid catabolism [28]. This observation may be related to differences in release kinetics and the bioaccessibility of tannic acid among formulations; uncoated or powdered forms may release tannic acid more readily in the upper gastrointestinal tract, allowing interactions with dietary proteins and digestive enzymes [9,18]. However, it is also possible that the LPTA formulation delivered a higher effective dose of tannic acid compared with the other formulations, contributing to the observed effects. As no in vitro or in vivo release kinetics data were generated in this study, these proposed explanations regarding release characteristics and dose differences remain hypothetical and require further validation through direct experimental evidence. In contrast, coated and granular formulations may exert weaker effects on protein utilization efficiency due to delayed or heterogeneous release characteristics, and potentially lower delivered doses of active tannic acid.

With respect to lipid metabolism, although tannic acid is generally reported to exert lipid-lowering effects [29], the LGTA group showed a significant increase in serum triglyceride (TG) levels, which is inconsistent with previous reports [30,31]. This discrepancy suggests that formulation characteristics and/or dose differences may modulate the metabolic effects of tannic acid. It is hypothesized that the granular formulation may alter the release kinetics and site of action of tannic acid in the gastrointestinal tract, thereby influencing lipid metabolism; however, as no in vitro or in vivo release kinetics data were generated in this study, direct evidence for this mechanism is currently lacking, and this hypothesis requires further investigation. In contrast, TG levels were not significantly altered in the LPTA and LCTA groups, suggesting that, under the present experimental conditions, powdered and coated formulations did not exert measurable adverse effects on lipid metabolism.

With respect to antioxidant status, differences were observed among formulations. The LCTA group showed significantly increased CAT and SOD activities, suggesting activation of the antioxidant defense system, consistent with previous reports [32]. However, MDA levels were also significantly elevated in this group, indicating increased lipid peroxidation and suggesting a potential pro-oxidant effect. Previous studies have reported that tannic acid can be metabolized by gut microbiota into low-molecular-weight phenolic metabolites, which may participate in Fenton-type reactions and promote oxidative processes under certain conditions [33,34], although this mechanism was not directly verified in the present study. In addition, increased *IL-6* expression in the LCTA group may be associated with enhanced oxidative stress. In contrast, MDA levels were not elevated in the LPTA group and instead showed a decreasing trend, suggesting a more stable redox homeostasis. The LGTA group exhibited an intermediate antioxidant profile between LCTA and LPTA. These differences may reflect variations in release kinetics, site of action, and/or the actual dose of tannic acid delivered by each formulation. However, in the absence of direct in vitro or in vivo release kinetics data, this interpretation remains speculative and requires experimental verification.

With respect to intestinal morphology and barrier integrity, all formulations resulted in decreased villus height compared with the control group, consistent with the astringent nature of tannic acid [35,36]. This observation indicates that tannic acid, regardless of formulation, exerted a generally negative effect on intestinal epithelial structure. However, the LPTA group exhibited the highest villus height-to-crypt depth ratio (VH/CD) among the treatment groups, suggesting a relatively more favorable balance between epithelial injury and renewal compared with the other formulations. It is important to emphasize, however, that this relative improvement in VH/CD should not be interpreted as evidence of net intestinal protection, particularly given the concurrent decreases in villus height across all groups, the downregulation of *Claudin-1* and *Occludin* expression, and the upregulation of *NF-κB*. The powdered formulation may act primarily in the proximal small intestine, potentially inducing compensatory epithelial responses, whereas coated and granular formulations may exert effects in more distal intestinal segments. However, as no in vitro or in vivo release kinetics data were generated in this study, this interpretation regarding site-specific action remains speculative and requires confirmation through direct measurements of tannic acid concentrations in different intestinal segments. The observed differences may also reflect variations in the actual dose of tannic acid reaching different intestinal segments. Overall, while LPTA may have exerted a less detrimental effect on intestinal morphology compared with the other formulations, the present data do not support a conclusion of superior intestinal protective effects. Further studies, including functional permeability assays, are needed to better evaluate the net impact of these formulations on intestinal health and barrier function.

With respect to tight junction proteins, all treatments showed significant downregulation of *Claudin-1* and *Occludin* expression. As these proteins are critical components of epithelial tight junctions, their downregulation is generally interpreted as reduced barrier integrity rather than barrier enhancement. This finding differs from some previous reports that observed barrier-protective effects of tannins in certain experimental contexts [37,38], suggesting that the effects of tannic acid on intestinal barrier function may be context-dependent, varying with factors such as dose, formulation, exposure duration, and the specific intestinal segment examined [30,32]. Notably, *ZO-1* expression in the LPTA group was comparable to that of the CON group, with no significant difference detected between these two groups, while *MUC-2* showed an increasing trend. These observations might indicate a potential compensatory effect through enhancement of the mucus barrier, which may partially offset the changes in tight junction protein expression observed in other treatment groups.

With respect to immune regulation, the present study showed that all tannic acid treatments were associated with upregulation of *NF-κB* mRNA expression, accompanied by reduced expression of *TNF-α* and *IL-10*. This pattern does not conform to the classical inflammatory response profile, in which *NF-κB* activation typically increases pro-inflammatory cytokines. Several factors may explain this discrepancy. The functional outcome of *NF-κB* activation depends on its dimeric composition: p50/p50 homodimers can repress transcription, whereas p65/p50 heterodimers activate it. In addition, *NF-κB* simultaneously induces negative feedback regulators such as IκBα, which may terminate inflammatory responses. Moreover, the measured mRNA levels represent a single time point, and post-transcriptional mechanisms could uncouple mRNA from protein levels. Tannic acid has also been reported to exert context-dependent effects on *NF-κB* signaling [11]. Therefore, while the data suggest complex immune modulation, these explanations remain speculative and require further investigation. Notably, *IL-6* expression was significantly increased only in the LCTA group, suggesting that the coated formulation may have a greater propensity to induce pro-inflammatory responses. Encapsulation systems have been shown to exhibit pH-dependent release behavior, limiting release under gastric conditions while promoting release in the intestinal phase [39], which may deliver higher local concentrations of tannic acid to distal intestinal segments and elicit different mucosal immune responses compared with powdered formulations. However, direct evidence for this mechanism is currently lacking, and this interpretation remains hypothetical.

With respect to the gut microbiota, 16S rRNA sequencing revealed differences in microbial community structure among the three formulation groups. However, PERMANOVA analysis did not detect statistically significant differences in overall microbial community structure among groups (*p* = 0.152), indicating that the observed clustering represents formulation-dependent trends rather than significant community-level shifts. Alpha diversity analysis showed that the Chao1 index was not significantly altered in the LPTA group, whereas the Shannon index increased, suggesting improved community evenness while maintaining richness. In contrast, the Chao1 index decreased in the LCTA and LGTA groups, indicating that these formulations may have influenced microbial community structure and could be associated with reduced colonization resistance; however, as colonization resistance was not directly measured in this study, this interpretation remains speculative [40].

To further explore the potential functional characteristics of the gut microbiota, Tax4Fun was applied to predict KEGG functional profiles based on 16S rRNA sequencing data. At KEGG Level 2, the predicted functional composition was mainly dominated by carbohydrate metabolism, membrane transport, replication and repair, amino acid metabolism, translation, and energy metabolism. Compared with the control group, tannic acid supplementation resulted in a lower predicted relative abundance of carbohydrate metabolism-related functions, as well as varying decreases in membrane transport, replication and repair, and amino acid metabolism-related functions. In contrast, translation- and energy metabolism-related functional categories showed increasing trends. At KEGG Level 3, the predicted abundances of transporter proteins, ABC transporters, DNA repair and recombination proteins, purine metabolism, and pyrimidine metabolism-related functions showed decreasing trends following tannic acid supplementation, whereas tRNA biosynthesis and two-component system-related functions showed increasing trends. Overall, Tax4Fun prediction suggested that tannic acid supplementation may influence the potential functional composition of the gut microbiota, particularly pathways related to nutrient metabolism, transport, and cellular maintenance. However, the predicted functional profiles among LCTA, LPTA, and LGTA groups showed largely comparable patterns, suggesting limited formulation-specific effects on microbial functional potential under the present experimental conditions. It is important to emphasize that Tax4Fun provides computational predictions based on taxonomic composition rather than direct measurements of microbial function; therefore, these predicted functional profiles require validation through metagenomic, metabolomic, or transcriptomic analyses in future studies.

At the phylum level, the LPTA and LGTA groups showed increased relative abundance of *Firmicutes* and decreased *Bacteroidota*, consistent with previous studies [9]. However, coating or microencapsulation may modify interactions between tannic acid and the gut microbiota by delaying its release in the upper gastrointestinal tract, thereby attenuating its regulatory effects on phylum-level composition [41].

At the genus level, both the LCTA and LPTA groups showed enrichment of *Bifidobacterium*, a genus known for its ability to metabolize tannins and produce short-chain fatty acids (SCFAs) and bioactive phenolic compounds [18]. However, the increased abundance of *Bifidobacterium* should be interpreted cautiously, as beneficial microbial changes were not consistently accompanied by improvements in physiological or intestinal barrier parameters (e.g., villus height, tight junction protein expression). Moreover, as SCFA concentrations were not directly measured in this study, the potential for enhanced SCFA production remains speculative and requires verification through targeted metabolite analysis. The LPTA group was additionally enriched in *Alistipes*, a genus associated with anti-inflammatory properties and intestinal homeostasis [38]. *Negativibacillus*, which was enriched in the LPTA group, belongs to the family *Ruminococcaceae* and has been associated with butyrate production and maintenance of intestinal homeostasis [42]. *Enterorhabdus*, a genus within the family *Eggerthellaceae*, was more abundant in the LCTA group; members of the *Eggerthellaceae* family have been reported to metabolize polyphenolic compounds [43]. *Micrococcales*, an order of *Actinobacteria* detected at low abundance, may contribute to the metabolic versatility of the gut microbiota, although its specific functional roles require further investigation. Although the LCTA group also exhibited enrichment of *Bifidobacterium*, the reduction in Chao1 index suggests that coated tannic acid may exert inhibitory effects on microbial richness, possibly due to localized release and elevated tannic acid concentrations in distal intestinal regions. The LGTA group exhibited intermediate microbial alterations between the other treatments. Overall, while Tax4Fun prediction and genus-level compositional changes suggest potential functional shifts, the biological significance of these changes remains to be determined through integrated metabolomic and functional assays, as direct measurements of SCFAs, phenolic metabolites, digestive enzyme activities, and nutrient digestibility were not performed in the present study.

From a practical standpoint, the selection of commercial tannic acid formulations for poultry production should consider the trade-offs among biological efficacy, production cost, and manufacturing complexity. The powdered formulation (LPTA) appeared to exhibit a relatively balanced biological profile under the present experimental conditions, and its simpler manufacturing process likely results in lower production costs compared with coated or granular products [44]. However, its rapid release characteristics may lead to high localized concentrations in the gastrointestinal tract, potentially impairing palatability and inducing stress responses under certain conditions [9]. In contrast, coated (LCTA) and granular (LGTA) formulations offer advantages in controlled release and targeted delivery due to their more complex processing [9,14], but these benefits are accompanied by higher production costs and, in the case of the coated product, potentially pro-inflammatory effects as suggested by the increased *IL-6* expression observed in the LCTA group. Therefore, the choice of formulation should be guided by specific production goals: powdered formulations may be suitable for cost-sensitive applications where moderate efficacy is acceptable, whereas coated or granular formulations may be preferred when targeted delivery and sustained release are prioritized, provided that the additional costs are justified by the production context. Ultimately, the optimal formulation will depend on the specific production system, economic considerations, and desired outcomes.

Overall, differences were observed among the three tannic acid formulations in yellow-feathered broilers, which might be associated with variations in release kinetics, site of action, and/or actual delivered dose. Under the present conditions, LPTA exhibited a more balanced profile in terms of protein utilization, redox homeostasis, and gut microbiota modulation, whereas the coated formulation may be associated with elevated oxidative stress and the granular formulation showed altered lipid metabolism. However, these findings require further validation. A key limitation is that the three products were compared at equal commercial inclusion rates; thus, the observed differences may reflect variations in actual tannic acid content and bioavailability rather than formulation alone. Specifically, the observed biological differences cannot be unequivocally attributed to formulation characteristics because formulation type and actual tannic acid dose were inherently confounded. This is the principal limitation of the study. Furthermore, no release kinetics data were generated, so statements regarding release characteristics remain hypothetical. Future studies incorporating dose–response analyses and release kinetics measurements are needed to elucidate the underlying mechanisms.

## 5. Conclusions

In conclusion, the three commercial tannic acid products differed in their biological effects in yellow-feathered broilers when compared at an equal commercial inclusion rate of 250 mg/kg. It should be emphasized that the present findings are applicable to comparisons among commercial products rather than to formulation characteristics alone, because the actual active tannic acid doses differed among treatments. The powdered product (LPTA) appeared to exhibit a relatively balanced profile in terms of antioxidant status, intestinal barrier function, and gut microbiota modulation under the present experimental conditions. However, it is important to emphasize that the products differed in active tannic acid content (50%, 65%, and 75% for coated, powdered, and granular, respectively), and thus the actual delivered doses were not equal (125, 162.5, and 187.5 mg/kg). Therefore, the observed product-specific differences may reflect variations in both formulation characteristics and actual active dose, and cannot be attributed exclusively to formulation. Future studies incorporating dose–response designs with equalized active ingredient levels are needed to further elucidate the mechanisms underlying these differences and to optimize the application of tannic acid products in poultry production.

## Figures and Tables

**Table 1:** Composition and Nutritional Levels of Experimental Diets.

Items	1–21 d/Content (%)	22–42 d/Content (%)
Corn	51.635	57.395
Soybean meal	24.9	19.3
wheat flour	10	10
Cotton seed meal	5	5
Corn protein meal	4	4
Limestone powder	1.6	1.4
Calcium hydrogen phosphate I	0.9	0.98
Sodium chloride	0.27	0.28
L-Lysine Sulfate	0.53	0.58
Bentonite	0.425	0
Soybean oil	0	0.4
Premix ^1^	0.23	0.225
DL-methionine	0.17	0.15
Choline chloride	0.1	0.1
Benzoic acid	0.1	0
Dinitrotriamine premix	0	0.05
L-threonine	0.08	0.09
Maltose	0.04	0.03
Phytase	0.02	0.02
Total	100	100
Nutrient levels ^2^		
DM	87.65	87.26
Moisture	12.29	12.69
Ash	5.64	4.91
CP	21	19
EE	2.30	2.81
CF	3.18	2.95
NDF	8.42	8.15
ADF	3.67	3.40

^1^ The premix provides per kilogram of feed: Mg 100 mg, Zn 75 mg, Fe 75 mg, I 0.65 mg, Cu 8 mg, Se 0.35 mg, VA 9000 IU, VD_3_ 2000 IU, VE 20 IU, VK_3_ 3.1 mg, VB_1_ 1.2 mg, VB_2_ 6.0 mg, niacin 66 mg, pantothenic acid 10 mg, VB_6_ 2.6 mg, biotin 0.1 mg, folic acid 0.7 mg, VB_12_ 0.02 mg. ^2^ DM, dry matter; Moisture, moisture content; Ash, crude ash; CP, crude protein; EE, ether extract; CF, crude fiber; NDF, neutral detergent fiber; ADF, acid detergent fiber; With the exception of metabolizable energy, which is a calculated value, all nutrient level data are determined by direct measurement.

**Table 2:** Primer Design for This Experiment.

Genes	Forward—Sequence (from 5′ to 3′)	Reverse—Sequence (from 3′ to 5′)	Genbank Number
*β-actin*	ACTCTGGTGATGGTGTTAC	GGCTGTGATCTCCTTCTG	NM 205518.2
*Claudin-l*	CACTGCCACTCCCTGATGTT	ACCGGTGACAGACTGGTTTC	NM_001013611.2
*Occhudin*	GCATCACAGCCGCCCT	TTACAAAATGCCTTCCCAAAAAGC	NM_205128.1
*MUC-2*	GTGCTCTGAACAGCAATAAC	AACTCCATTGTAATCCCCAC	XM_015279046.4
*TNF-α*	AGAGGCGTTTCCTCGTC	AACCATCAAGAGACCCTGAG	XM_046900549.1
*IL-10*	ATGGATGAGAACGGGATCTA	TTGGAAGGGGAGAAAACAAA	NM_001004414.4
*IL-6*	TCATCCTCCGAGACTTTACT	CCGAACTAAAACATTCAGGC	NM_204628.2
*IFN-γ*	GCTCCCGATGAACGACTTGA	TGCTGAGGTATTGCTGATGG	NM_034476.3
*ZO-1*	CTTCAGGTGTTTCTCTTCCTCCTC	CTGTGGTTTCATGGCTGGATC	XM_040706827.2
*NF-κB*	TCAACGCAGGACCTAAAGACAT	GCAGATAGCCAAGTTCAGGATG	XM_015285417.3

**Table 3:** Effects of Tannic Acid in Different Dosage Forms on the Growth Performance of 42-Day-Old Yellow-Feathered Broilers.

Item	CON	LCTA	LPTA	LGTA	SEM	*p*-Value
IBW/g (1–21 d)	35.66 ± 0.8 ^a^	35.92 ± 0.91 ^a^	35.64 ± 1.08 ^a^	36.14 ± 0.64 ^a^	0.17	0.73
FBW/g (1–42 d)	1434.14 ± 48.78 ^a^	1454.46 ± 35.13 ^a^	1460 ± 61.91 ^a^	1434.86 ± 74.97 ^a^	11.14	0.80
1–42 d						
ADG g/d	33.3 ± 1.16 ^a^	33.78 ± 0.82 ^a^	33.91 ± 1.47 ^a^	33.3 ± 1.79 ^a^	0.27	0.80
ADFI g/d	69.95 ± 2.43 ^a^	70.65 ± 1.9 ^a^	71.73 ± 2.99 ^a^	71.23 ± 3.08 ^a^	0.52	0.68
FCR %	2.1 ± 0.05 ^a^	2.09 ± 0.02 ^a^	2.12 ± 0.03 ^a^	2.14 ± 0.05 ^a^	0.008	0.16

IBW, initial body weight (g; day21); FBW, final body weight (g; day 42); ADG, average daily gain (g/day); ADFI, average daily feed intake (g/day); FCR, feed conversion ratio (feed/gain). Identical letters denote no significant difference.

**Table 4:** Effects of Tannic Acid in Different Dosage Forms on Serum Biochemistry in 42-Day-Old Yellow-Feathered Broiler Chickens.

Item	CON	LCTA	LPTA	LGTA	SEM	*p*-Value
TP (g/L)	22.60 ± 1.37 ^c^	35.45 ± 1.32 ^ab^	34.27 ± 1.69 ^b^	37.44 ± 1.42 ^a^	1.78	<0.01
ALB (g/L)	11.53 ± 1.06 ^c^	12.85 ± 0.52 ^b^	13.56 ± 0.47 ^ab^	14.24 ± 0.24 ^a^	0.34	0.005
GLOB (g/L)	12.46 ± 0.20 ^c^	23.14 ± 1.29 ^a^	19.98 ± 0.87 ^b^	22.86 ± 0.91 ^a^	1.32	<0.01
TC (mmol/L)	3.14 ± 0.14 ^ab^	3.32 ± 0.10 ^a^	3.03 ± 0.03 ^b^	3.17 ± 0.07 ^ab^	0.04	0.04
TG (mmol/L)	0.36 ± 0.02 ^bc^	0.42 ± 0.01 ^ab^	0.32 ± 0.09 ^c^	0.50 ± 0.02 ^a^	0.02	0.007
UREA (mmol/L)	0.49 ± 0.04 ^a^	0.49 ± 0.02 ^a^	0.37 ± 0.02 ^b^	0.46 ± 0.04 ^a^	0.02	0.004

TP, total protein (g/L); ALB, albumin (g/L); GLOB, globulin (g/L); TC, total cholesterol (mmol/L); TG, triglycerides (mmol/L); UREA, urea (mmol/L). Data are presented as mean ± standard deviation (SD). SEM, standard error of the mean. a–c Within a row, different superscript letters indicate significant differences among groups (*p* < 0.05). Different superscript letters denote significant differences (*p* < 0.05); identical letters denote no significant difference.

## Data Availability

The data presented in this study are available on request from the corresponding authors.
